# Deferral of Non-Emergency Cardiovascular Interventions Triggers Increased Cardiac Emergency Admissions—Analysis of the COVID-19 Related Lockdown

**DOI:** 10.3390/ijerph192416579

**Published:** 2022-12-09

**Authors:** Dominik Felbel, Sascha d’Almeida, Manuel Rattka, Stefanie Andreß, Kathrin Reischmann, Benjamin Mayer, Armin Imhof, Dominik Buckert, Wolfgang Rottbauer, Sinisa Markovic, Tilman Stephan

**Affiliations:** 1 Department of Cardiology, Angiology, Pneumology and Intensive Care Medicine, University of Ulm, 89081 Ulm, Germany; 2 Institute for Epidemiology and Medical Biometry, Ulm University, 89075 Ulm, Germany

**Keywords:** COVID-19, lockdown, cardiac event, non-emergency, emergency, admission, lockdown strategies, deferral

## Abstract

Background: Data on the relation between non-emergency and emergency cardiac admission rates during the COVID-19 lockdown and post-lockdown period are sparse. Methods: Consecutive cardiac patients admitted to our tertiary heart center between 1 January and 30 June 2020 were included. The observation period of 6 months was analyzed in total and divided into three defined time periods: the pre-lockdown (1 January–19 March), lockdown (20 March–19 April), and post-lockdown (20 April–30 June) period. These were compared to the reference periods 2019 and 2022 using daily admission rates and incidence rate ratios (IRR). Results: Over the observation period from 1 January to 30 June, cardiac admissions (including non-emergency and emergency) were comparable between 2019, 2020, and 2022 (n = 2889, n = 2952, n = 2956; *p* = 0.845). However, when compared to the reference period 2019, non-emergency admissions decreased in 2020 (1364 vs. 1663; *p* = 0.02), while emergency admissions significantly increased (1588 vs. 1226; *p* < 0.001). Further analysis of the lockdown period revealed that non-emergency admissions dropped by 82% (IRR 0.18; 95%-CI 0.14–0.24; *p* < 0.001) and 42% fewer invasive cardiac interventions were performed (*p* < 0.001), whereas the post-lockdown period showed a 52% increase of emergency admissions (IRR 1.47; 95%-CI 1.31–1.65; *p* < 0.001) compared to 2019. Conclusions: We demonstrate a drastic surge of emergency cardiac admissions post-COVID-19 related lockdown suggesting that patients who did not keep their non-emergency appointment had to be admitted as an emergency later on.

## 1. Introduction

Since the beginning of the COVID-19 outbreak, there have been many reports on potential deteriorating direct and indirect effects of the pandemic on people’s health [1]. In several medical specialties, reduced hospital admission rates were recorded for patients suffering from acute disease and prolonged delay times from symptom onset to first medical contact have been registered [2,3]. However, especially in patients suffering from cardiovascular disease representing a leading cause of death and acquired disability worldwide [4], timely medical care is essential to avoid detrimental outcomes [5,6]. It has been shown that declined admission numbers of patients with an acute cardiac event are associated with worse clinical presentation, elevated plasma levels of cardiac biomarkers at admission, and higher short-term mortality [2]. Moreover, a recent study demonstrated that even the deferral of non-emergency cardiac interventions due to prioritization of healthcare services has been linked to a significantly worse clinical outcome [7].

Nowadays, existing evidence led to rising concern that COVID-19-related lockdown and healthcare reorganization resulting in significant reduced emergency and non-emergency treatment numbers could negatively impact patients’ long-term outcomes [7,8,9]. While most of the previous investigations focused on admission rates of patients suffering from an acute cardiac event in the early phase of the pandemic [7,10,11], data on non-emergency cardiac presentations, and particularly on the relation between non-emergency and emergency admission rates over the time course of the COVID-19 pandemic, including the reopening phase as well, are sparse [12].

However, such knowledge will be of great importance in the ongoing and future pandemics as it could highlight the possible collateral effects of a lockdown strategy and reorganization of healthcare.

In the present study, we analyzed the time-dependent course of non-emergency and emergency admissions of cardiac patients treated at our high-volume tertiary hospital center before, during, and after COVID-19-related lockdown, for the first time.

## 2. Methods

This monocentric retrospective comparative study included consecutive patients suffering from cardiac diseases admitted to our university hospital center between 1 January 2020 and 30 June 2020 (study group). The Ulm University Medical Center is a tertiary care center in the south of Germany representing an area inhabited by approximately 200,000 people and treats most cardiac emergencies in the region. The study period comprises the first national lockdown in Germany between 21 March, when rigorous public health measures came into effect, and 20 April, when public opening was partly initiated again. During the lockdown period, non-emergency cardiovascular interventions were postponed according to current recommendations of cardiologic societies in order to meet the higher demand for hospital beds for COVID-19-infected patients. Emergency cardiac interventions were carried out as usual besides the treatment of patients with COVID-19.

Admissions were subdivided into scheduled and emergency admissions and admission numbers were compared to a control period in the previous year (1 January–30 June 2019; control group). Emergency admission was defined as every unplanned admission due to an acute cardiac event via our emergency department, while non-emergency admissions comprised all patients admitted for scheduled cardiac interventions. Transfers from external hospitals or readmissions within 24 h were not considered in admission analysis.

To further investigate the effect of the different phases of the COVID-19 pandemic ((1) before, (2) during, and (3) after the first national lockdown), we divided the observation period of 6 months into three heterogenous time frames: the pre-lockdown period, lasting from 1 January to 19 March (78 days), the 31-day long lockdown period from 20 March to 19 April, as well as the post-lockdown period, lasting from 20 April to 30 June (72 days). Admission numbers amongst the three previously defined periods in 2020 were compared to each other. Additionally, admission numbers of the pre-lockdown, lockdown, and post-lockdown period were compared to their respective seasonal control group from 2019.

Moreover, the numbers of cardiac interventions including coronary angiography, percutaneous coronary interventions (PCI), electrophysiological procedures, non-surgical valve interventions, and heart device implantations were analyzed. Rates of non-emergency admissions and procedures such as electrophysiological procedures, heart valve interventions, and heart device were calculated for weekday only.

In a further step, data from 2019 and 2020 were compared to 2022 in order to better evaluate the results as well as to assess a possible recovery of the health care system from the COVID-19 pandemic. The study was conducted according to the guidelines of the Declaration of Helsinki. Ethical review and approval were waived for this study, due to the study design and the fact that only admission numbers and no patient specific data were analyzed.

## 3. Statistical Analysis

Continuous variables are presented as median and interquartile range (IQR) or mean and standard deviation (SD) as appropriate. Continuous variables for two groups were compared with the Student’s *t*-test or the unpaired U-test. Continuous variables for three groups were compared using the Kruskal–Wallis test. The Shapiro–Wilk test was used to test for normal distribution. Categorical data are presented as absolute and relative frequencies and compared with the χ^2^-test. Daily admission numbers were compared between the three periods using ANOVA.

Crude incidence rates per day and incidence rate ratios including 95% confidence intervals (CI) comparing the study period with the control periods were calculated using Poison regression to model the number of non-emergency and emergency admissions as well as cardiac interventions per day. A two-sided *p*-value < 0.05 was considered to indicate statistically significant. Statistical analyses were performed with IBM’s SPSS version 26 and SAS version 9.4 under Windows.

## 4. Results

During the study period in 2020, a total of 2952 patients were admitted to the Department of Cardiology at the University Medical Center, Ulm, Germany. Out of these, 1364 patients (46.2%) were scheduled for non-emergency interventions while 1588 patients (53.8%) were admitted due to an acute cardiac event.

**Figure 1:**
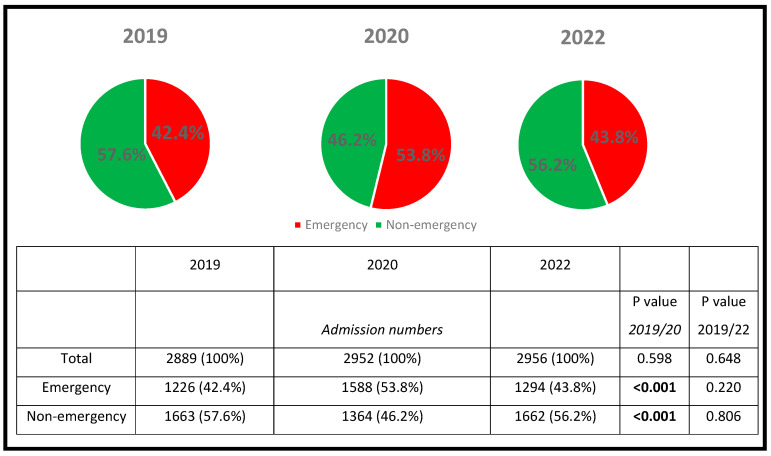
Comparison of the number of cardiac emergency and non-emergency admissions in 2019, 2020 and 2022 from 1 January and 30 June.

During the control period in 2019, a comparable number of patients (N = 2889) were admitted to our hospital center, but non-emergency interventions were significantly higher (57.6% vs. 46.2%; *p* < 0.001) and emergency admissions were less frequent (42.4% vs. 53.8%; *p* < 0.001) as displayed in Figure 1.

In 2019, 13.1 ± 4.7 patients were admitted as non-emergency per day, while 10.7 ± 5.5 patients were admitted per day in 2020 (*p* < 0.001). In contrast, the number of emergency admissions significantly increased from 6.8 ± 2.9 patients per day in 2019 to 8.7 ± 3.6 patients per day in 2020 (*p* < 0.001) (Table 1).

### 4.1. Comparison of Non-Emergency Admission Numbers Peri-Lockdown

**Figure 2:**
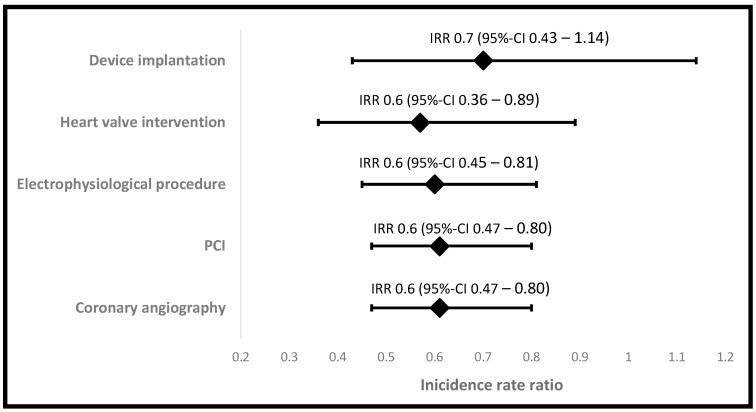
Incidence rate ratios of cardiac procedures during the lockdown period compared to the reference period in 2019. The *x*-axis displays the IRR of each intervention type during the lockdown period (20 March–19 April) in 2020 compared to the corresponding period in 2019. IRR: incidence rate ratio; CI: confidence interval; PCI: percutaneous coronary intervention.

During the lockdown period in 2020, 2.4 ± 2.8 patients per day with cardiac disease were admitted as non-emergency to our Department of Cardiology. In comparison to the same time period in 2019 (13.4 ± 4.1 non-emergency admissions per day), non-emergency admissions were significantly lower (IRR 0.18; 95%-CI 0.14–0.24; *p* < 0.001) (Table 2 and Figure 2). Additionally, the number of non-emergency admissions during the lockdown period was significantly decreased when compared to the pre- (13.1 ± 4.0 patients per day) and post-lockdown (11.6 ± 4.3) periods in 2020 as well (lockdown vs. pre-lockdown period: IRR 0.19; 95%-CI 0.14–0.24; *p* < 0.001 and lockdown vs. post-lockdown period: IRR 0.22; 95%-CI 0.16–0.28; *p* < 0.001, respectively).

**Figure 3:**
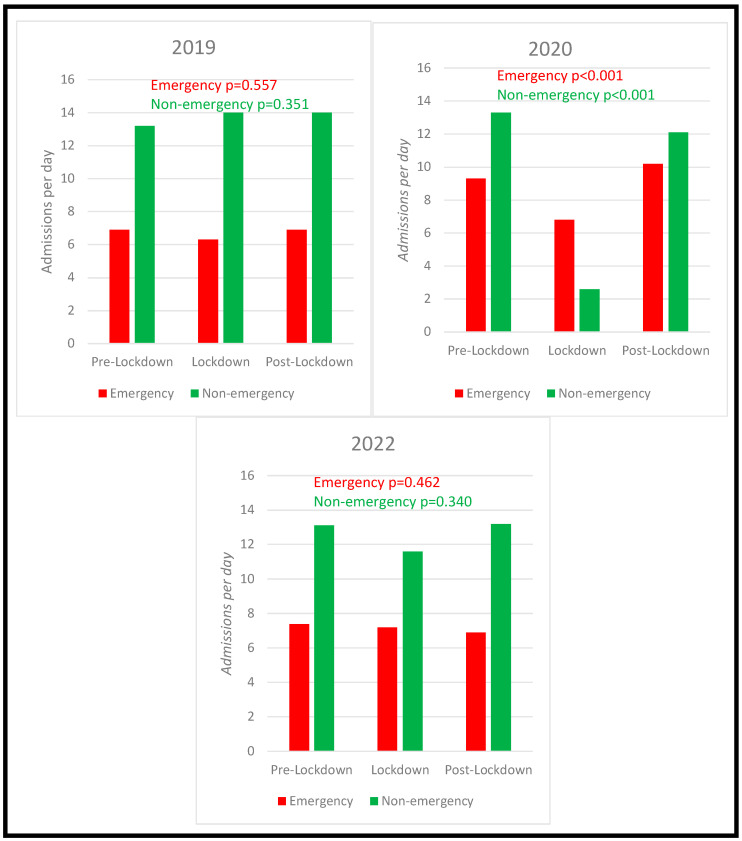
Mean admission numbers per day during each period in 2019, 2020 and 2022 stratified by emergency and non-emergency. Pre-Lockdown (1 January to 19 March), Lockdown (20 March–19 April) and Post-Lockdown (20 April to 30 June).

In the post-lockdown period, the numbers of non-emergency admissions did not differ significantly if compared to the respective seasonal control group in 2019 (IRR 0.91; 95%-CI 0.81–1.02; *p* = 0.107). Within the control year 2019, non-emergency admissions were similarly distributed across the three periods (pre-lockdown period: 13.2 ± 4.3 non-emergency admissions, lockdown period 13.4 ± 4.1 non-emergency admissions, and post-lockdown 12.9 ± 5.5 non-emergency admissions; *p* = 0.351) (Figure 3).

### 4.2. Comparison of Emergency Admission Numbers Peri-Lockdown

During the lockdown period in 2020, 6.7 ± 2.9 cardiac emergencies per day were admitted to our emergency department. The number of emergency admissions during the national lockdown was similar to the corresponding period in 2019 (IRR 1.09; 95%-CI 0.90–1.32; *p* = 0.380) and significantly lower compared to the 2020 pre-lockdown period (IRR 0.84; 95%-CI 0.72–0.98; *p* = 0.02). Remarkably, we observed a significant increase in emergency admissions in the post-lockdown period compared to the pre-lockdown period (IRR 1.25; 95%-CI 1.12–1.39; *p* < 0.001) and the lockdown period (IRR 0.67; CI 0.58-0.78; *p* < 0.001). Additionally, an increase of emergency admission numbers post-lockdown 2020 was seen if compared to the corresponding period in the previous year (IRR 1.47; CI 1.31–1.65; *p* < 0.001). Table 2 presents the crude incidence rates (per day) for non-emergency and emergency hospital admissions within the three study periods in 2019 and 2020 as well as the incidence rate ratios for the corresponding group comparisons.

### 4.3. Invasive Cardiac Procedures

An amount of 9.9 ± 6.1 invasive cardiac interventions were performed per day during lockdown 2020. Compared to the corresponding period in 2019, we observed a reduction by 43% of invasive procedures undertaken (17.4 ± 8.9; *p* < 0.001). In detail, there was a significant reduction of 42% for coronary angiography (IRR 0.61; *p* < 0.001), 42% for percutaneous coronary interventions (IRR 0.61; *p* < 0.001), 43% for heart valve interventions (IRR 0.57; *p* = 0.014), and 37% for electrophysiological procedures (IRR 0.60; *p* < 0.001) as well as a trend towards fewer device implantations (21%; IRR 0.70; *p* = 0.146). In the post-lockdown period, the total number of invasive cardiac procedures tended to increase when compared to their corresponding period in 2019 (20.2 vs. 17.6 procedures per day; *p* = 0.149). IRR and daily numbers of invasive cardiac procedures are displayed in Figure 2, in Table 1, Table 2 and Table 3 as well as in the Supplementary.

**Table 3:** Incidence rate ratios and daily numbers of hospital admissions and invasive cardiac procedures between the post-lockdown period 2020 and the corresponding period 2019 (20 April–30 June).

	Post-Lockdown Period 2020	Control Period 2019
	20 April–30 June	20 April–30 June
Total admissions		15.7 ± 8.6
N per day	18.3 ± 8.1	
*p* value	0.055	
Emergency admissions		
N per day	10.2 ± 3.8	6.9 ± 2.8
IRR (95%-CI)	1.47 (1.31–1.65)	
*p* value	**<0.001**	
Non-emergency admissions		
N per day	11.6 ± 4.3	12.9 ± 5.5
IRR (95%-CI)	0.91 (0.81–1.02)	
*p* value	0.107	
Total cardiac invasive procedures		17.6 ± 10.6
N per day	20.2 ± 10.7	
*p* value	0.149	
Coronary angiography		6.8 ± 3.8
N per day	7.4 ± 3.9	
*p* value	0.914	
Percutaneous coronary intervention		5.0 ± 3.1
N per day	4.9 ± 2.9	
*p* value	0.914	
Electrophysiological procedure		4.8 ± 2.9
N per day	7.1 ± 2.9	
*p* value	**<0.001**	
Valve interventions		3.1 ± 2.2
N per day	3.0 ± 2.0	
*p* value	0.852	
Device implantation		1.3 ± 1.1
N per day	1.8 ± 1.9	
*p* value	0.628	

IRR: incidence rate ratio; CI: confidence interval daily admission numbers are displayed by mean with standard deviation. Statistical significant *p*-values are displayed bold.

### 4.4. Comparison of Emergency and Non-Emergency Admission Numbers between 2019 and 2022

Between 1 January and 30 June 2022, a total of 2956 cardiac patients, consisting of 1294 emergency (43.8%) and 1662 non-emergency admissions (56.2%), were admitted to our hospital center. Over the observation period, the total admission rates were comparable between 2019, 2020, and 2022 (*p* = 0.845). Both the total number of patients (*p* = 0.648) and the ratio between emergency and non-emergency admissions (*p* = 0.220 and *p* = 0.806, respectively) were comparable to the year 2019 (Figure 1, Table 4). Furthermore, emergency and non-emergency daily admission rates were similarly distributed across the three study periods (*p* = 0.462 and *p* = 0.340, respectively) and were comparable to their corresponding time periods in 2019.

## 5. Discussion

This study analyzed incidence rates of both non-emergency and emergency admissions during COVID-19-related lockdown compared to the corresponding period in 2019, as well as the pre- and post-lockdown periods in 2020. Remarkably, we observed a significant decline in admission numbers of non-emergency cardiac patients during lockdown, while there was a significant surge in emergency admissions in the post-lockdown period. Simultaneously, there was a significant decrease by 43% of invasive cardiac procedures during the lockdown period 2020 when compared to the corresponding period 2019. Consequently, our results let us hypothesize that cardiac patients who did not make or did not keep their appointment during lockdown, were supposedly admitted as emergency cases later.

In the early phase of the pandemic, healthcare professionals already warned against the potential harmful effects of the pandemic on patients without COVID-19 [13]. It has been suggested that, amongst others, fear of COVID-19 might prevent patients from presenting for medical attention [14]. Up to now, there have been many reports on declining hospital admissions ranging from 27% [12] to 43% [11] during the ongoing pandemic, but the underlying reasons remain speculative [3]. Especially, patients with cardiovascular disease display a vulnerable population, since a delay in timely medical care prolongs the total ischemic time, thereby negatively affecting their prognosis [15]. Several studies demonstrated that during the COVID-19 outbreak the decline in admissions of emergency patients suffering from acute coronary syndrome was associated with a significantly prolonged time from symptom onset to first medical contact, potentially deteriorating the patients’ outcomes [16,17]. Moreover, the number of patients presenting with heart failure declined over the peak of the pandemic as well [7,9,18] and those who did present had more adverse outcomes [19]. Higher mortality rates were described for both in hospital [19,20,21] and even after discharge [8]. Similarly, out-of-hospital mortality rates increased for patients with pre-existing cardiovascular conditions over the course of the pandemic [12].

In our study, we observed that during the COVID-19-related lockdown admission numbers of patients suffering from an acute cardiac event dropped by 16.4% compared to the pre-COVID-19 period, thereby substantiating previous findings [22]. Several factors have been discussed to at least in part contribute to the absence of heart patients, amongst others, altruistic behavior to not overburden the hospital personnel, framing issues, and the influence of the media [2].

As expected, the number of non-emergency cardiac patients admitted to our center declined as well. The main cause was an anticipated demand surge for hospital beds of many hospitals that therefore reduced or postponed non-emergency admissions, which was the same for our center [13,23,24]. Hereby, deferral was applied for non-emergency interventions in patients with supposed stable disease in accordance with the current cardiologic societies’ recommendations. Furthermore, it has been suggested that some patients cancel their appointments because the fear of getting infected with SARS-CoV-2 steers them away from presenting at hospital [14,25].

In the post-lockdown period, daily non-emergency admission numbers did not differ significantly regarding the incidence rate ratio when compared to its corresponding period in 2019, suggesting a recovery of non-emergency admissions after the lockdown.

Nonetheless, compared to the pre-lockdown group, one would expect a shift from significantly lower admission numbers in the lockdown group to significantly higher admissions numbers in the post-lockdown group, since cancelled or postponed procedures presumably accumulate after lockdown. However, the final course of these “missing” non-emergency patients remains unclear. Death by out-of-hospital cardiac arrest, treatment in a different hospital or admission as a medical emergency could have contributed to this finding. In this context, a recent study observed a 52% increase in out-of-hospital cardiac arrest correlated to the COVID-19 pandemic as compared with 2019 [26].

As a consequence, we evaluated the incidence of medical emergency admissions during lockdown in 2020, the corresponding period in 2019, as well as before and after lockdown in 2020. Intriguingly, we demonstrated that admissions due to an acute cardiac event increased about 49% in the post-lockdown period compared to the lockdown period. Additionally, compared to the control group from 2019, the incidence of cardiac emergency admissions was significantly higher during the post-lockdown phase and increased by almost 52%, suggesting that indeed patients who did not keep their non-emergency appointment during lockdown might have been admitted by the emergency medical services due to an acute cardiac event later on. This thesis is supported by a previous study showing that deferred cardiac patients, despite being classified as postponable, were associated with increased emergency hospitalization and progression of symptoms within the first 365 days [27].

Furthermore, a recent study associated longer waiting times in patients with severe valvular disease with higher morbidity and mortality. Heart failure hospitalization was observed in 12% of patients with planned transcatheter aortic valve implantation after three months waiting time [28]. Moreover, about 7% of patients with indicated percutaneous mitral valve repair experienced heart failure hospitalization at 30 days [29].

In the 2022 period, we were able to show that both emergency and non-emergency daily admission rates and the ratio between emergency and non-emergency admissions were again comparable to 2019, indicating a recovery of the healthcare system. Data of 2022 ultimately underline our observation of a post-lockdown-related increase of emergency admissions; however, further studies reporting the follow-up of patients who did not keep their appointment or whose appointment was postponed are needed to verify this hypothesis.

The COVID-19 pandemic has led to a substantial reduction of invasive cardiac operations and interventions [7,18,30,31,32]. In our study, invasive cardiac interventions were reduced by 43%. The reduced performance rate is most likely to be explained by both the reduction of admission rates for acute cardiac disease and the postponement of non-emergency cardiac interventions during the initial phase of the pandemic as recommended by cardiological societies. In our study, cardiac interventions such as coronary angiographies, percutaneous coronary interventions, heart valve interventions, and electrophysiological procedures were reduced by about 40% compared to the reference period 2019.

Our data are consistent with other studies investigating performance rates of cardiac interventions during the early phase of the pandemic. A nationwide report from England showed a significant reduction of procedure rates in April 2020 of up to 41.2% for percutaneous coronary interventions, 35.4% for transcatheter aortic valve replacement, 88.8% for percutaneous ablation, and 52.8% for cardiac devices [32]. It should be noticed that a direct comparison may be impeded by nation specific health care strategies during the COVID-19 pandemic.

Interestingly, invasive cardiac procedures raised numerically by 15% in the post-lockdown period 2020 when compared to the reference period, suggesting that most deferred cardiac patients received their non-emergency cardiac intervention at a later date as emergency procedure. This rise did not reach statistical significance (*p* = 0.149), which might be explained by the fact that procedure numbers can only be increased to a certain degree due to limited catheter and personal capacities.

Our findings underline that strategies to manage patients with cardiovascular disease during the current and ongoing COVID-19 pandemic are essential. Research already tried to develop strategies to identify patients who are in a condition allowing them to safely defer non-emergency procedures; however, these strategies urgently need further development and revision [33]. Anyway, health care professionals should carefully consider postponing non-emergency cardiac interventions in the future management of the COVID-19 pandemic since this might result in avoidable dangers for heart patients.

## 6. Limitations

Our study has some limitations. This single center retrospective study carries all the inherent limitations of retrospective research. Due to the explorative and retrospective character of this study, our results have to be interpreted as hypothesis generating. Our results are based on admission numbers and do not include clinical follow-up. Therefore, the influence of other factors cannot finally be excluded. However, our data are in line with previous analyses.

## 7. Conclusions

This is the first study evaluating both incidence rate ratios of non-emergency and emergency patients admitted to a high-volume tertiary care center during COVID-19-related lockdown compared to an in-year and a seasonal control group. We demonstrated that while non-emergency admissions significantly declined during lockdown, there was a drastic surge of emergency cardiac admissions post-lockdown, suggesting that patients who did not keep their non-emergency appointment had to be admitted as an emergency at a later time. In 2022, non-emergency and emergency admission ratios recovered to the pre-COVID-19 pandemic period.

However, medical professionals should carefully balance the benefits and risks of postponing non-emergency interventions, even in the current and ongoing pandemic.

## Figures and Tables

**Table 1:** Comparison of the number of hospital admissions and invasive cardiac procedures per day in 2019 and 2020 (1 January–30 June).

	Year		
	2019	2020	
	N per Day	N per Day	*p*-Value
Type of admission			
Total admissions	15.9 ± 8.6	16.22 ± 8.5	0.598
Non-emergency admissions	13.1 ± 4.7	10.7 ± 5.5	**<0.001**
Emergency admissions	6.8 ± 2.9	8.7 ± 3.6	**<0.001**
Total invasive cardiac procedures	17.1 ± 9.7	18.1 ± 10.6	0.394
Coronary angiography	6.5 ± 3.6	6.76 ± 4.0	0.670
Percutaneous coronary intervention	4.5 ± 2.9	4.3 ± 2.7	0.901
Electrophysiological procedure	4.9 ± 2.6	5.9 ± 3.5	**0.013**
Valve intervention	2.8 ± 2.0	2.8 ± 1.9	0.968
Device implantation	1.4 ± 1.2	1.6 ± 1.7	0.830

Data are described as mean with standard deviation. Statistical significant *p*-values are displayed bold.

**Table 2:** Incidence rate ratios and daily numbers of hospital admissions and invasive cardiac procedures between the lockdown period 2020 and the reference period 2019 (20 March–19 April).

	Lockdown Period 2020	Control Period 2019
	20 March–19 April	20 March–19 April
Total admissions		
N per day	8.3 ± 4.7	16.2 ± 7.8
IRR (95%-CI)	0.61 (0.53–0.7)	
*p* value	**0.03**	
Emergency admissions		
N per day	6.7 ± 2.9	6.3 ± 2.9
IRR (95%-CI)	1.09 (0.89–1.32)	
*p* value	0.379	
Non-emergency admissions		
N per day	2.4 ± 2.8	13.4 ± 4.1
IRR (95%-CI)	0.18 (0.14–0.24)	
*p* value	**<0.001**	
Total cardiac invasive procedures		
N per day	9.9 ± 6.1	17.4 ± 8.9
*p* value	**<0.001**	
Coronary angiography		
N per day	3.7 ± 3.2	6.4 ± 3.2
IRR (95%-CI)	0.61 (0.47–0.8)	
*p* value	**<0.001**	
Percutaneous coronary intervention		
N per day	2.6 ± 1.7	4.5 ± 2.7
IRR (95%-CI)	0.61 (0.47–0.8)	
*p* value	**<0.001**	
Electrophysiological procedure		
N per day	3.2 ± 3.5	5.1 ± 2.3
IRR (95%-CI)	0.6 (0.45–0.81)	
*p* value	**<0.001**	
Heart valve intervention		
N per day	1.3 ± 1.5	2.3 ± 1.6
IRR (95%-CI)	0.57 (0.36–0.89)	
*p* value	**0.014**	
Device implantation		
N per day	1.5 ± 1.6	1.9 ± 1.2
IRR (95%-CI)	0.7 (0.43–1.14)	
*p* value	0.146	

IRR: incidence rate ratio; CI: confidence interval; daily numbers are displayed by mean with standard deviation. Statistical significant *p*-values are displayed bold.

**Table 4:** Comparison of the number of hospital admissions and invasive cardiac procedures per day in 2019 and 2022 (1 January–30 June).

	Year		
	2019	2022	
	N per Day	N per Day	*p*-Value
Type of admission			
Total admissions	15.9 ± 8.6	16.3 ± 8.0	0.656
Non-emergency admissions	13.1 ± 4.7	12.9 ± 4.4	0.661
Emergency admissions	6.8 ± 2.9	7.2 ± 2.5	0.220

Data are described as mean with standard deviation.

## Supplementary Materials

The following supporting information can be downloaded at: https://www.mdpi.com/article/10.3390/ijerph192416579/s1, Table S1: Incidence rate ratio of hospital admissions and invasive cardiac procedures during the lockdown period compared to the pre- and post-lockdown period. Table S2: Comparison of hospital admissions and invasive cardiac procedures between the pre-lockdown period 2020 and the corresponding period (1 Januray–20 March).
