# Supplementary material for: Deferral of Non-Emergency Cardiovascular Interventions Triggers Increased Cardiac Emergency Admissions—Analysis of the COVID-19 Related Lockdown
Source: Int J Environ Res Public Health. 2022 Dec 9;19(24):16579. doi: 10.3390/ijerph192416579 (PMC9778764; doi:10.3390/ijerph192416579)
Supplement: Supplementary file 1 [file ijerph-19-16579-s001.zip › ijerph-2054935-supplementary.pdf]

Supplements

Table S1. Incidence rate ratio of hospital admissions and invasive cardiac procedures during the lockdown period compared to the pre- and post-lockdown period.

Admission type	Pre-lockdown period 2020<br>1 January – 19 March	Post-lockdown period 2020<br>20 April – 30 June
Total admissions<br>IRR (95%-CI)<br>p value	 0.55 (0.49 – 0.62)<br>p<0.001	 0.52 (0.46 – 0.59)<br>p<0.001
Emergency admissions<br>IRR (95%-CI)<br>p value	 0.84 (0.72 – 0.98)<br>p=0.02	 0.67 (0.58 – 0.78)<br>p<0.001
Non-emergency admissions<br>IRR (95%-CI)<br>p value	 0.19 (0.14 – 0.24)<br>p<0.001	 0.22 (0.16 – 0.28)<br>p<0.001
Coronary angiography<br>IRR (95%-CI)<br>p value	 0.53 (0.43 – 0.64)<br>p<0.001	 0.53 (0.43 – 0.64)<br>p<0.001
Percutaneous coronary intervention<br>IRR (95%-CI)<br>p value	 0.59 (0.46 – 0.74)<br>p<0.001	 0.36 (0.29 – 0.45)<br>p<0.001
Electrophysiological procedure<br>IRR (95%-CI)<br>p value	 0.5 (0.39 – 0.66)<br>p<0.001	 0.45 (0.35 – 0.59)<br>p<0.001
Heart valve intervention<br>IRR (95%-CI)<br>p value	 0.42 (0.28 – 0.62)<br>p<0.001	 0.46 (0.31 – 0.69)<br>p<0.001
Device implantation<br>IRR (95%-CI)<br>p value	 0.79 (0.52 – 1.21)<br>p=0.288	 0.73 (0.48 – 1.12)<br>p=0.149

CI: Confidence interval.
Incidence rate ratios are displayed for the lockdown period compared to the pre-lockdown and the post-lockdown period.

Table S2. Comparison of hospital admissions and invasive cardiac procedures between the pre-lockdown period 2020 and the corresponding period (1 January – 20 March)

	Pre-lockdown 2019<br>1 January – 19 March	Control period 2020<br>1 January – 19 March	
Type of admission	*n per day*	*n per day*	*P-Value*
Total admissions	15.9 ±8.9	17.5 ±8.5	0.262
Emergency admission	6.9 ± 3.0	8.2 ± 3.1	**0.007**
Non-emergency admission	13.2 ±4.3	13.1 ±4.0	0.974

Daily admission numbers are displayed by mean with standard deviation.
